# Beckwith-Wiedemann Syndrome With Severe Relapsing Hypoglycemia After the Neonatal Period: A Case Report and a Literature Review

**DOI:** 10.7759/cureus.57588

**Published:** 2024-04-04

**Authors:** Hossam A Aldosari, Ameera F Alghamdi

**Affiliations:** 1 Pediatrics, King Fahd Military Medical Complex, Dhahran, SAU

**Keywords:** beckwith-wiedemann syndrome, omphalocele chromosome 11p15, paternal disomy, hyperinsulinemic hypoglycemia, bws

## Abstract

Beckwith-Wiedemann syndrome (BWS) is a rare genomic imprinting disorder that affects multiple systems. Major features can manifest as large birth weight, anterior abdominal wall defects, macroglossia, hyperinsulinism, organomegaly hemihypertrophy, and renal abnormalities. Characteristic facies manifested as midface hypoplasia, infraorbital creases, facial nevus simplex, and anterior linear ear lobe creases/posterior helical ear pits, with a predisposition to tumor development. This case report describes a Saudi infant born at 38+5 weeks gestation via elective cesarean section to a 33-year-old G3P2+0 mother, with a family history of type 1 diabetes and Down syndrome. Prenatal ultrasound revealed an anterior abdominal wall defect. Postnatally, the infant exhibited macrosomia, macroglossia, and omphalocele. Genetic testing confirmed paternal disomy of the imprinted region in 11p15.5. The infant underwent successful omphalocele repair but experienced respiratory distress, and seizures on the third day of life. Intubation, ventilation, and antiepileptic treatment were initiated. Subsequent investigations revealed right upper lobe collapse, neonatal seizures on electroencephalogram (EEG), and thin corpus callosum on magnetic resonance imaging (MRI). Feeding difficulties led to elective partial glossectomy at two months of age. During her hospital stay two days post surgery, the infant developed persistent hypoglycemia requiring high glucose infusion rates. Extensive endocrine evaluation revealed high insulin and cortisol levels. Subcutaneous octreotide was administered with minimal response. After 15 days of careful glucose tapering, the infant's blood glucose stabilized, reaching feeding targets. The patient was discharged with follow-up appointments. This comprehensive case highlights the complexity of managing severe relapsing hypoglycemia in an infant with BWS.

## Introduction

Beckwith-Wiedemann syndrome (BWS) is an overgrowth disorder characterized by segmental overgrowth, enlarged organs, macroglossia, abdominal wall defects, and ear and renal abnormalities [1,2]. However, these presentations can vary significantly, and not all individuals with the syndrome exhibit all these clinical features. The first documented case of BWS was described by Beckwith in 1963 whereas after one-year Wiedemann documented cases similar to one described previously [3,4]. Current estimates suggest that the prevalence of BWS is one in 10,000-21,000 births [5]. One notable aspect is its association with congenital hyperinsulinism. In infants with BWS, the frequency of hypoglycemia ranges from 30% to 50% [6,7]. Most affected infants are asymptomatic, and hypoglycemia typically resolves within the first three days of life. However, less than 5% may experience persistent hypoglycemia beyond the neonatal period, necessitating continuous feeding or even a partial pancreatectomy. The cause of hypoglycemia in BWS is not fully understood, but evidence points to hyperinsulinemia as a major contributing factor. Recent advances in genetics have identified specific genes on chromosome 11p15, including ABCC8 and KCNJ11, as being involved in the abnormal regulation of insulin in BWS. Understanding the genotype-phenotype relationship in BWS, particularly in cases requiring a partial pancreatectomy, may shed light on the molecular basis of hypoglycemia in this syndrome. Comprehensive evaluations of genotype and phenotype are crucial for gaining insights into the mechanisms underlying hypoglycemia in BWS [8,9]. We present a case of BWS that experienced rebound prolonged hypoglycemia after the neonatal period.

## Case presentation

**Figure 1:**
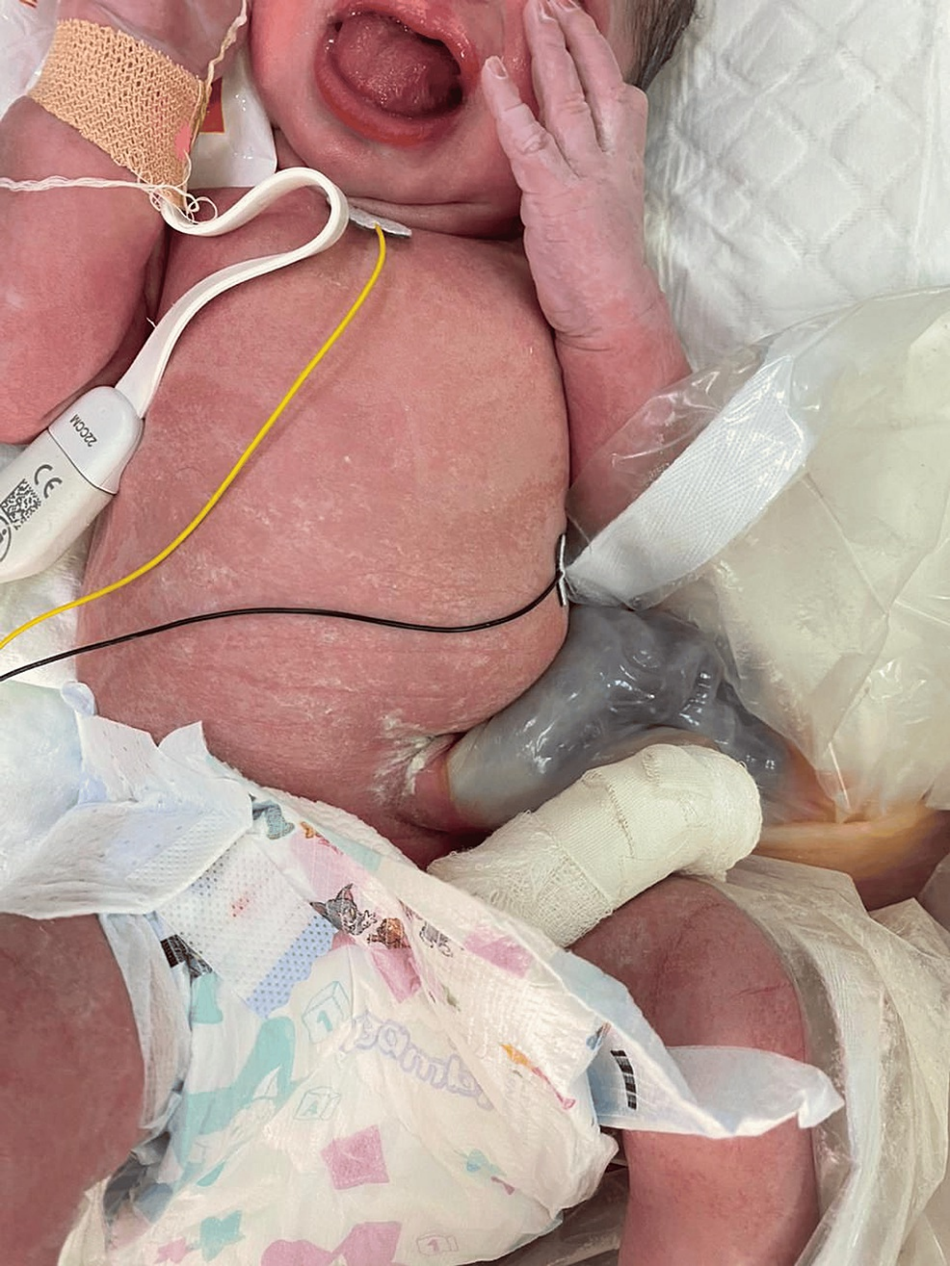
Upon delivery showing macroglossia and umbilical cord containing bowel intact sac

We present the case of a two-month-old Saudi infant female delivered at 38+5 weeks via elective cesarean section. The infant is the third daughter of a 33-year-old G3P2+0 mother, both parents non-consanguineous and without significant medical history. During prenatal screening at 25 weeks of gestation, an ultrasound revealed a single viable fetus with an anterior abdominal wall defect, characterized by bowel protrusion, intra-abdominal liver and stomach, and polyhydramnios. Therefore, neonatology and pediatric surgery teams were involved in the delivery. The infant, weighing 3.875 kg (98th percentile) and measuring 49 cm in height (73rd percentile) with a head circumference of 34 cm (80th percentile), displayed features suggestive of BWS, including macrosomia, macroglossia, depressed nasal bridge, and omphalocele (Figure 1).

The protruding sac was managed with wet gauze and a plastic sheet, and the infant was transferred to the neonatal intensive care unit (NICU). And planned for omphalocele repair the next day. Cord blood gas analysis revealed a pH of 7.304, pCO2 of 54 mmHg, HCO3 of 22.5 mmol/L, Lactate 1.9 mmol/L, base excess of 0.4 mmol/L, Sodium (Na) 140 mmol/L, Potassium (K) 3.9 mmol/L, and random glucose level of 0.7 mmol/L (12.6 mg/dL). In response to these findings, the patient received intravenous administration of 10% dextrose solution at a rate of 2 mL/kg. Subsequent random blood glucose readings were obtained every three hours, revealing the following values: 3 pm: 2.8 mmol/L (50.4 mg/dL), 6 pm: 3.1 mmol/L (55.8 mg/dL), and 9 pm: 2.6 mmol/L (46.8 mg/dL). Complete blood count showed: Hemoglobin (HGB): 19.4 g/dL, Hematocrit (HCT): 63.4%, Platelet Count (PLT): 257 * 10^3/μL, White Blood Cell Count (WBC): 10.4, Absolute Neutrophil Count (ANC): 4.86 * 10^3/μL, Neutrophil Percentage (N%): 46.6%, Lymphocyte Percentage (L%): 36.8%, and Monocyte Percentage (M%): 11.2%.

Head ultrasonography was unremarkable except for a thin corpus Callosum. Abdominal ultrasound showed bilateral Grade 1 hydronephrosis. Echocardiography revealed a small atrial septal defect (ASD) and a small patent ductus arteriosus (PDA), Whole exome sequencing (WES) revealed no indication of deletion or duplication in the BWS/SRS critical region by multiplex ligation-dependent probe amplification (MLPA). Methylation analysis showed hypermethylation in the differentially methylated region of H19/GF2: IG-DMR in combination with hypomethylation of the KCNQ1OT1: TSS-DMR in the BWS/SRS critical region in 11p15.5. These methylation alterations suggested a paternal disomy of at least the imprinted region in 11p15.5 (UPD (11) pat), confirmed by independent MLPA analysis.

**Figure 2:**
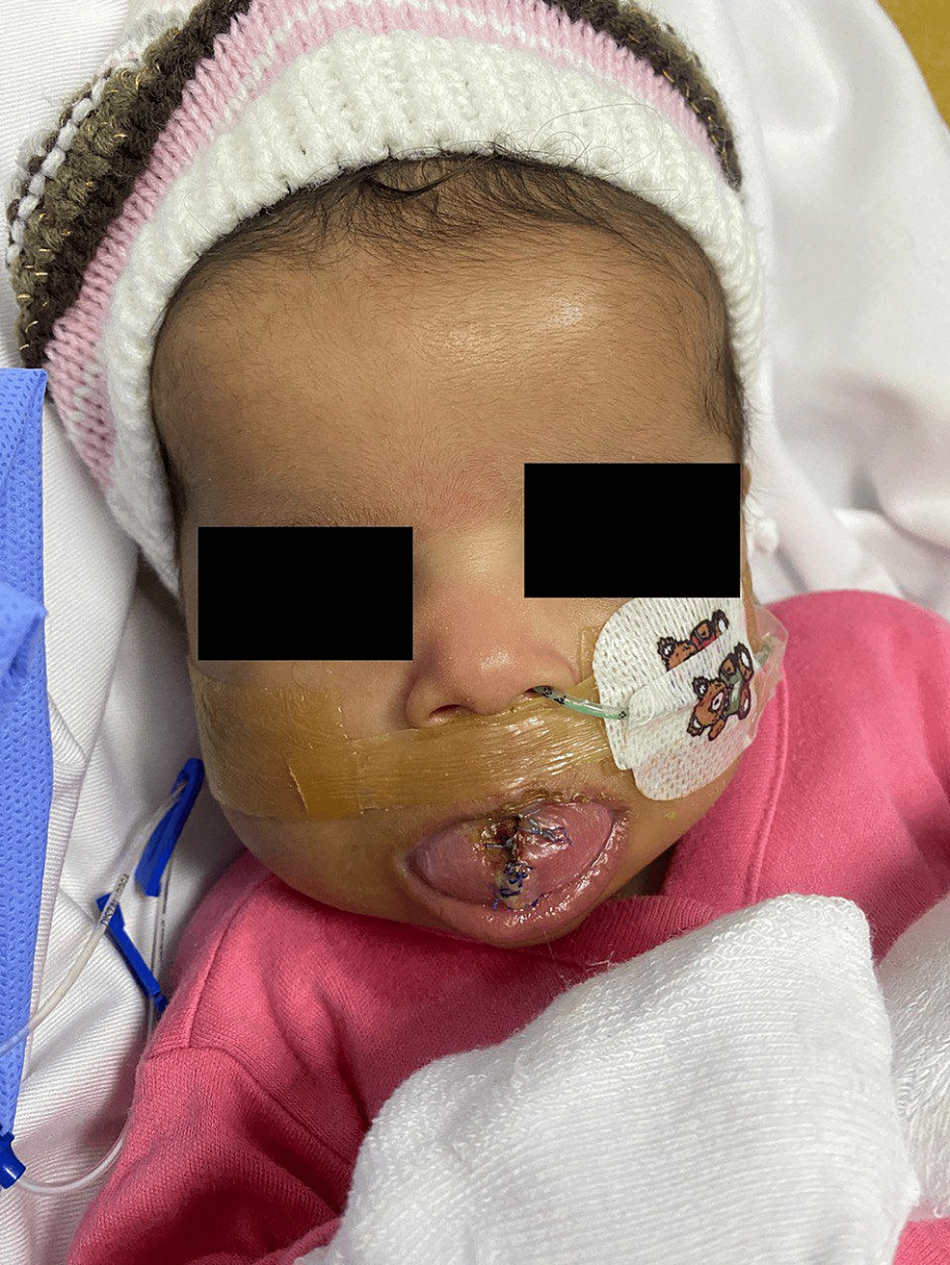
Post-partial glossectomy surgery

On the second day of life, the patient underwent omphalocele repair without complications. However, on the third day, the patient experienced stridor, desaturation (down to 50%), bradycardia (down to 70 b/min), and generalized tonic movements. Random blood sugar was 6.2 mmol/L (111.6 mg/dL). The patient was intubated and connected to a conventional ventilator. After intubation, heart rate and oxygen saturation improved, and the patient self-extubated the next day. An electroencephalography study revealed multifocal spikes and waves correlating with neonatal seizures, while brain magnetic resonance imaging (MRI) showed a thin corpus callosum. Gradual feeding progress was made until the infant achieved full feeds on the seventeenth day of life, with no hypoglycemic or abnormal movement episodes upon discharge. At home, the baby appeared well, displaying activity and normal movements. The only reported concern from the mother was feeding difficulties due to a large tongue. Elective admission for partial glossectomy occurred at one month and 26 days of age, with a smooth surgery and subsequent transfer to the Pediatric Intensive Care Unit (PICU) (Figure 2).

On the second day post-admission at two months of age, the infant experienced frequent hypoglycemic episodes, with random blood sugar readings ranging from 0.6 to 1.7 mmol/L (10.8 mg/dL - 30.6 mg/dL) over 24 hours. Despite receiving three boluses of IV D10% and a very high Glucose Infusion Rate (GIR) reaching 17 mg/kg/min with D25%, there was no improvement. Critical samples were sent for various tests that was available at our laboratory, revealing abnormal results including low random blood sugar, elevated insulin level, and other metabolic markers (Table 1).

**Table 1:** Summary of critical sample results

parameter	Value	Reference limits
Random blood sugar	2.45 mmol/L	3.6 - 11.0
insulin	16.1 μU/mL	0.87 - 20.44
C peptide	3.01 ng/mL	0.78 - 5.19
Serum ketons	Less than 0.1	<0.6
Cortisol	545.6 nmol/L	-
Growth Hormone	2.01 ng/mL	-
Ammonia	50.46 Umol/L	18 - 72
Lactate	1.65 mmol/L	0.5 - 2.2

The baby was transferred back to the ward and was initiated on nasogastric feeding, but persistent hypoglycemia persisted. our endocrinology team recommended subcutaneous octreotide administration, as first-line diazoxide was unavailable. given subcutaneously with an initial dose of 5/mcg/kg/day given as 10 mcg every eight hours, with a gradual increment of 5-10 mcg/kg/day until reached to high dose of 32 mcg/kg/day, given as 40 mcg every six hours, However, this treatment was discontinued after five days of initiation due to a lack of response. The infant remained in the hospital for 15 days, during which a gradual tapering of high dextrose infusion was necessary to prevent rebound hypoglycemia. By the 15th day, blood glucose levels were maintained between 4 and 6 mmol/L (72-108 mg/dL) with no further hypoglycemic episodes.

Upon discharge, the baby reached the feeding target of 120 ml every three hours with the addition of one spoon of Carboch. Additionally, the infant was referred to a higher center for further evaluation by specialists in Oncology and Genetics.

## Discussion

The case presented describes a complex medical scenario involving an infant with BWS, omphalocele, and subsequent complications including respiratory distress, seizures, and severe early and late hypoglycemia. Clinical findings suggested in the present case are consistent with those reported in BWS. However, prolonged hypoglycemia is a significant finding as the prevalence of symptomatic hypoglycemia is only reported in 5% of the patients [6,7]. Moreover, the development of severe relapsing hypoglycemia after the recovery of an early hypoglycemic episode following a stressful event has been rarely reported in the literature before, and Hussain et al., in their study reported the histological findings from pancreas obtained by pancreatectomy [10]. Their findings showed a marked proliferation of endocrine tissues which formed irregular nodules. Furthermore, they showed that ATP-sensitive potassium (KATP) channel defect was responsible for hypoglycemia [10]. In the present case, hypoglycemia developed on the first day after birth. This is consistent with previous findings, which also reported instant development of hypoglycemia. Adachi et al. reported that their patient developed hypoglycemia within 1.5 hours after birth [11]. Similarly, a case report by Shah et al. found that their patient developed hypoglycemia within 30 minutes after birth [12]. Hypoglycemia is a significant concern in the management of BWS. According to Elliot et al. who investigated 74 cases of BWS, 63% of cases developed hypoglycemia [6]. In the present case, the patient showed abnormal features during pregnancy such as bowel protrusion, intra-abdominal liver and stomach, and polyhydramnios. This was suggestive of post-delivery complications; therefore, as performed in our case, a multidisciplinary care approach involving neonatology and pediatric surgery teams should be involved. However, such symptoms are not manifested in all cases. For example, Hussain et al. and Adachi et al. did not report complications at birth; however, patients developed BWS symptoms postnatally [10,11].

Among various genetic overgrowth syndromes, BWS is the most prevalent one [13]. In the present case, genetic testing revealed paternal uniparental disomy (UPD) in the imprinted region 11p15.5, confirming the diagnosis of BWS. The genetic components involved in BWS are very complex. Although alterations in various genetic bases can lead to BWS, the most common is an abnormal expression of genes in the p15.5 region of chromosome 11 [14]. Approximately 20% of individuals with BWS exhibit a sporadic somatic mosaic pattern of paternal UPD that affects at least the 11p15.5 region. In this scenario, the maternal p15.5 region of the chromosome is lost and replaced by a duplicated segment from the paternal chromosome. This alteration leads to an imbalance in gene expression favoring cell proliferation [15]. 

In the present case, a partial glossectomy was performed at two months of age. This is consistent with findings from previous cases as well. A case presented by Ulrich also reported that a patient had to undergo a partial glossectomy due to difficulty in drinking and feeding [5]. In the present case, patient developed one episode of transit hypoglycemia at one day of age with complete recovery and then at the age of two months patient admitted electively for partial glossectomy, during her hospital stay the infant was euglycemic until after the surgery when she then developed relapsing persistent severe hypoglycemia that could most probably been triggered by the stress of the surgery, In this case Two hypothesis can be thought of, first one is that patient was having persistent mild hypoglycemic episodes at home that was not shown clinically at home which explains not seeking medical advice, but what counteract this hypothesis is that our patient was active and thriving well and was euglycemic until 1 day prior to surgery when admitted electively for her partial glossectomy, which support our second hypothesis as hypoglycemia could most probably been triggered by the stress of the surgery, and as despite receiving high doses of IV dextrose fluids, the patient’s condition did not improve. In cases of BWS, IV dextrose can help stabilize blood glucose concentrations; however, this approach is not always effective. A study by Güemes et al. reported that in diazoxide unresponsive patients, blood glucose levels were maintained after infusing high-concentration dextrose along with glucagon and octreotide [16].

In cases of severe hypoglycemia, partial pancreatectomy can be considered. However, no exact guidelines exist regarding the extent of removing the pancreas in order to control hypoglycemia. A study by Laje et al. reported that all cases showed improvement in hypoglycemia after pancreatectomy [15]. However, Güemes et al. reported the use of sirolimus, an mTOR inhibitor to avoid pancreatectomy [16]. To investigate and compare the findings of published cases of BWS that developed hypoglycemia, a search was carried out in PubMed and Google Scholar. The keyword combination used was “Beckwith-Wiedemann Syndrome” AND “hypoglycemia.” A total of 11 studies were included in this literature review. Apart from one case, all cases were of infants. Table 2 shows the descriptive details of the previous studies.

**Table 2:** Descriptive details of previously published studies

Author and Year	Study Design	Participants	Major Findings	Timing and pattern of hypoglycemia	Hypoglycemia Treatment
Hussain et al., (2005) [10]	Case study	Newly born Baby BW= 3.8 kg	Hypoglycemia, hemihypertrophy, umbilical hernia, ear lobe crease, macroglossia.	Persistent hypoglycemia on the first day of life required a pancrectomy. At 14 months of age, recurrence of persistent hypoglycemia due to long fasting.	Partial pancreatectomyand octreotide Resolved after the last episode at 14 months
Adachi et al., (2013) [11]	Case study	Female infant; BW= 3.78 kg	Hypoglycemia observed after 1.5 hours after birth.	Persistent hypoglycemia 1.5 hours after birth.	Dizoxide and Octreotide Resolved at 3 months of age
Laje et al., (2013) [15]	Retrospective study	4 patients; 3 females, 1 male	Hypoglycemia Diffuse endocrine proliferation. One patient had pancreatoblastoma.	Profound hypoglycemia since birth	Pancreatectomy Resolved at 1-12 months of age
Al-Zubeidi et al., (2014) [17]	Case series	2 patient; 2 females;	Delayed hypoglycemia due to hydrocotrisone, macroglossia, hemihypertrophy, omphalocele	Persistent hypoglycemia at first 24 hours and eleventh day of life	Octerotide and Diazoxide Resolved at 2 - 6.5 years of age
Zarate et al., (2014) [18]	Case series	2 infants	Transient and refractory hypoglycemia.	Persistent hypoglycemia observed during the neonatal period Multiple persistent episodes during the first year of life	Octreotide Resolved at 17 months
Güemes et al., (2016) [16]	Case report	1-month-old female	Hypoglycemia, macroglossia, ear creases, enlarged kidneys, umbilical hernia.	Severe persistent hypoglycemia at the first day of life until 19 months	Dextrose (IV), glucagon,octreotide, Mtor inhibitor-Sirolimus Resolved after 19 months
Kalish et al., (2016) [19]	Retrospective study	28 children	Hypoglycemia, macrosomia, ear crease, macroglossia, hemihypertrophy, organomegaly, KATP channels mutation	Severe hypoglycemia at birth refractory to Diazoxide.	Subtotal pancreatectomies for 50% of children
Shah et al., (2020) [12]	Case study	Female; BW=3.7 kg	Hypoglycemia, umbilical hernia, macroglossia, ear crease. Hirschprung's disease.	Severe hypoglycemia 30 mins after birth controlled after surgery	Diazoxide and hydrochlorothiazide Resolved at 6 months of age

## Conclusions

In conclusion, this case highlights the complex management of BWS. A relapsing hypoglycemia after the neonatal period in BWS should be considered in all infants particularly those subjected to stress during or after surgery or any other form of stress as most probably it can trigger developing persistent hypoglycemia. The management of hypoglycemia poses a significant challenge. In the present case, genetic testing revealed paternal disomy in an infant, leading to postoperative complications including respiratory distress, seizures, and persistent hypoglycemia. Despite challenges, a 15-day hospital stay resulted in stabilized blood glucose levels, improved feeding, and resolution of hypoglycemic episodes.
